# In vitro bonding strength of denture teeth to denture base in CAD/CAM-milled, 3D-printed and conventional manufacturing processes

**DOI:** 10.1007/s00784-024-06099-y

**Published:** 2024-12-07

**Authors:** Marcel Löscher, Sebastian Hahnel, Reinhold Lang, Martin Rosentritt

**Affiliations:** Department of Prosthetic Dentistry, UKR University Hospital Regensburg, 93042 Regensburg, Germany

**Keywords:** Denture base, Denture tooth, TCML, Aging, 3D printing, Milling, CAD/CAM, Denture, Bonding

## Abstract

**Objectives:**

To investigate the survival rates and fracture resistance of dentures made from different teeth (milled, 3D-printed, fabricated), bases (milled, 3D-printed, pressed) and bonding combinations.

**Materials and methods:**

Specimens (11 groups, *n* = 8 per group) were fabricated from combinations with a denture tooth (anterior tooth 21) and a denture base material. The groups consisted of combinations of teeth (6x), denture base materials (5x) and adhesive bonding options (4x). The teeth were printed, milled or prefabricated. The denture base was produced conventionally or was milled or 3D-printed. Two dentures were milled from one industrially produced block. The dentures were subjected to thermal and mechanical loading (TCML) and subsequent fracture test. Statistics: ANOVA, Bonferroni-test, Kaplan-Meier survival, Pearson correlation; α = 0.05.

**Results:**

Mean loading cycles varied between 221,869 (8), 367,610 (11), 513,616 (6) 875,371 (3) and 9,000,030 (4). ANOVA revealed significant (*p* ≤ 0.001) different surviving cycles. Log Rank test showed significantly (*p* < 0.001) different loading cycles. Fracture force after TCML varied between 129.8 +/- 97.1 N (3) and 780.8 +/- 62.5 N (9). ANOVA comparison revealed significant (*p* < 0.001) different fracture loadings between the individual systems. Correlation was found between fracture force and loading cycles (0.587, *p* < 0.001).

**Conclusions:**

Different survival rates and fracture forces were found for dentures made of different teeth (milled, 3D-printed, prefabricated), bases (milled, 3D-printed, pressed) and bonding combinations. Milled, pressed and prefabricated systems provided longer survival and fracture force than the other tested systems.

**Clinical relevance:**

Optimal tooth-base combinations can help to produce a denture that is stable and resistant during clinical application.

## Introduction

Computer aided design and computer aided manufacturing (CAD/CAM) offers an efficient and cost-effective way to fabricate complete dentures [1, 2]. Digitally fabricated prostheses show similar accuracy [3], finer surfaces [4], lower weight [5], and better fit [6] compared to conventionally fabricated dentures. In addition, CAD/CAM dentures are well accepted by patients and fewer appointments with the dentist are required [7, 8].

A basic distinction for the CAD/CAM fabrication of dentures can be made between additive and subtractive manufacturing processes. Additive systems enable fast and material-saving production, while subtractive production using 5-axis milling machines also achieves high precision and consistent material properties thanks to industrially produced blanks [9–11]. In contrast to conventional methods, in both standard digital processes teeth and denture base are separately produced and are later joined together [12]. Combinations of the different fabrication methods are possible and the denture may benefit from the combinations of individual material properties. Therefore, besides the fracture strength of the denture, the bond between denture tooth and base is of major importance for the stability and longevity of the dentures [13]. However, due to various combinations of denture base, teeth and adhesive bonding, different bonding properties may be produced, implying differences in the performance of the bonding during clinical service. To avoid joining denture teeth and denture base, two-colored milling blanks (Ivotion, Ivoclar, Liechtenstein) have been introduced, which allow milling of the complete denture including teeth from a single blank.

As static bonding tests (e.g. according to DIN ISO/TS 19736; Bonding test between polymer teeth and denture base materials) allow a first assessment of the basic strength of the bond, only aging tests under dynamic load, possibly in combination with thermocycling, allow an estimation of the clinical performance [14]. To estimate the service life and clinical performance of a prosthesis, all specimens were aged in a thermal cycle and mechanical load test (TCML) and subsequently loaded to fracture. The hypothesis of this in-vitro study was that dentures made from different teeth (milled, 3D-printed, fabricated), bases (milled, 3D-printed, pressed) and bonding combinations have different in-vitro survival rates and fracture resistance. Conventional dentures were used as a control.

## Materials and methods

**Fig. 1:**
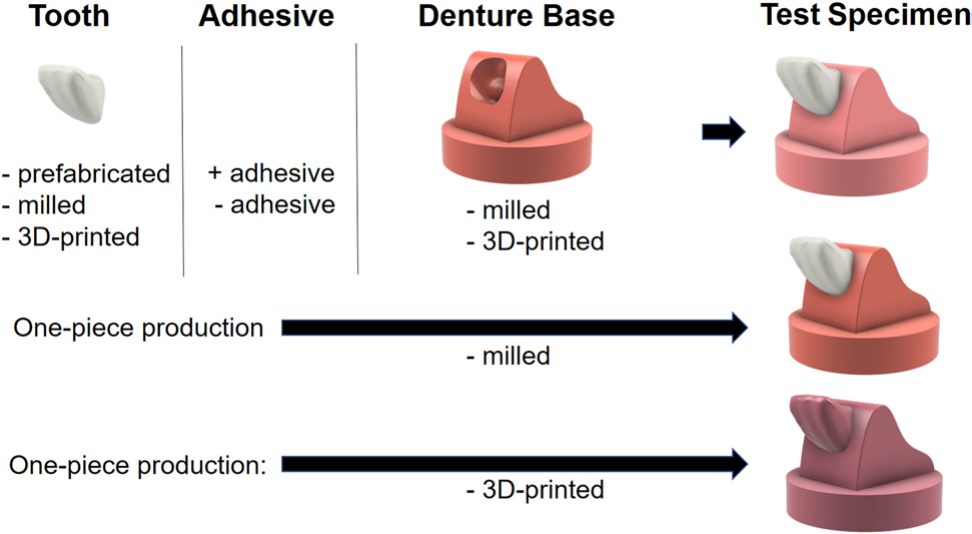
Diagram of the specimen and fabrication

Identical specimens were fabricated from combinations with a denture tooth (anterior tooth 21¸ design: Vionic Vigo Anterior R47; VITA Zahnfabrik, G; 90-98Wt% polymethyl methacrylate, 2-9Wt% silicon dioxide, 0–1 Wt% pigments) and a denture base material (11 groups with *n* = 8 per group). Teeth were mounted in an angle of 135 °C to vertical direction to simulate loading situation between upper and lower jaw. The groups consisted of different combinations of teeth (6x), denture base materials (5x) and adhesive bonding options (4x) (Fig. 1). The teeth were used in either 3D-printed, milled or prefabricated versions. The denture base was either produced conventionally or was milled or 3D-printed. In addition, dentures, which were milled from one industrially produced block, were examined (Table 1). Identical dimensions of teeth and denture base ensured a consistent contact surface. In all settings, a standardized bonding area of 75.3 mm^2^ (reference prefabricated tooth) was produced. Therefore, for the 3D-printed and milled teeth, the design of the prefabricated reference teeth was adopted accordingly (Fig. 1).

**Table 1:** Overview of the groups with denture tooth, adhesive and denture base materials (materials, manufacturers)

No.	Denture tooth	Adhesive	Denture base
1	Vionic Vigo (Vita Zahnfabrik)	Vionic Bond (Vita Zahnfabrik)	Vionic Base Disc (Vita Zahnfabrik)
2	Vionic Vigo (Vita Zahnfabrik)	-	ProBase Cold (Ivoclar)
3	Vionic Vigo (Vita Zahnfabrik)	Vionic Bond (Vita Zahnfabrik)	Denture Base Resin (Formlabs)
4	Vionic Vigo (Vita Zahnfabrik)	Denture Base Resin (Formlabs)	Denture Base Resin (Formlabs)
5	Vionic Dent Disc (Vita Zahnfabrik)	Vionic Bond (Vita Zahnfabrik)	Vionic Base Disc (Vita Zahnfabrik)
6	Vionic Dent Disc (Vita Zahnfabrik)	Vionic Bond (Vita Zahnfabrik)	Denture Base Resin (Formlabs)
7	Denture Base Resin (Formlabs)	-	Denture Base Resin (Formlabs)
8	Denture Teeth Resin (Formlabs)	Vionic Bond (Vita Zahnfabrik)	Denture Base Resin (Formlabs)
9	Ivotion Disc (Ivoclar)	-	Ivotion Disc (Ivoclar)
10	Ivotion Dent Disc (Ivoclar)	Ivotion Bond (Ivoclar)	Ivotion Base Disc (Ivoclar)
11	Denture Teeth Resin (Formlabs)	Denture Base Resin (Formlabs)	Denture Base Resin (Formlabs)

The 3D-printed parts (teeth and bases) were produced in a Form 3B+ (Formlabs Dental, USA; layer thickness 50 μm, base: 0° to the building platform, tooth: 45° to the building platform, base-tooth combination: 0° to the building platform) and then treated in a pre-wash (1 min 96% Isopropylalcohol) and a main wash (10 min 96% Isopropylalcohol, Form Wash, Formlabs Dental, USA). The final curing was carried out in an 85% glycol bath for 60 min at 80 °C (Form Cure, Formlabs Dental, USA) after short air drying.

The bonding (Denture Base Resin, Formlabs Dental, USA) procedure of the teeth was performed immediately after the main wash. The objects were air-dried. Liquid resin was applied with a microbrush and the tooth was positioned in the base. Fixation was achieved by irradiation with a polymerization lamp (Elipar S10, 3 M Espe, USA) for 15 s, followed by final curing (Form Cure, Formlabs Dental, USA; 60 min, 80 °C, Glycol 85%).

Parts (teeth and bases) to be milled were produced using the CORiTEC 350 Pro+ (imes-icore GmbH, G; wet milled, milling speed 25000 rpm, milling bur 2.5 mm) in a 5-axis milling process. All milled and 3D-printed surfaces, were sandblasted (50 μm Al_2_O_3_; 3 bar) and bonded using an adhesive (Vionic Bond, VITA, Germany; Ivotion Bond, Ivoclar, FL). The bonding was applied and cured according to the manufacturer’s instructions in a pressure vessel (Ivotion Bond: Polymat, Reitel, G, 15 min, 50 °C, 3 bar, 10 min processing time; Vionic Bond: Polymat, Reitel, G, 30 min, 55 °C, 2 bar, 30 min processing time; .

The completely milled test specimen group (Ivotion, Ivoclar, FL) was produced using a five-axis milling machine (PrograMill PM7, Ivoclar, FL).

To obtain a hollow mold for the cold-curing resin reference (ProBase Cold, Ivoclar, FL), a complete 3D-printed test specimen was molded with silicone (Turbosil, Klasse 4 Dental GmbH, G). Then, the tooth was fixed in the hollow mold and coated with monomer (ProBase Cold, Ivoclar, FL). The mixed denture base material (ratio of 15 g polymer to 10 mL monomer) was poured into the silicone mold and cured for 15 min at 40 °C/6 bar in a pressure vessel (Futuramat, Schütz-Dental).

**Fig. 2:**
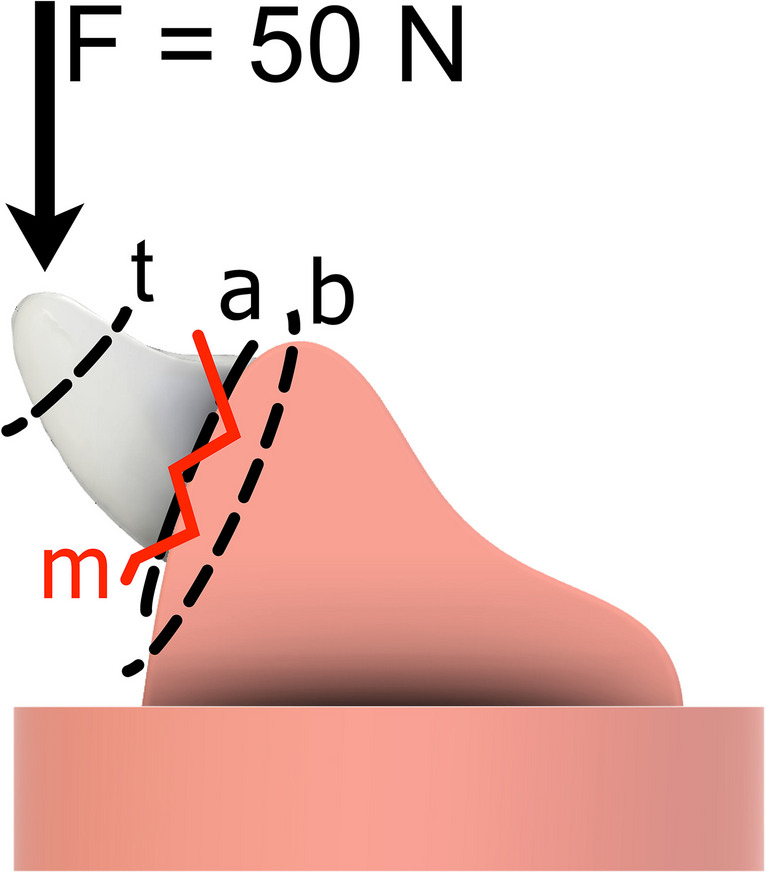
Specimen loading (scheme) and classification of fracture patterns (failure of the denture tooth (t), failure of the adhesive between base and tooth (**a**), fracture of the denture base (**b**), or mixed failure (m = a + b + t)

Eight of the dentures in each group were subjected to thermal and mechanical loading (TCML). For this purpose, the prosthesis was mounted in a chewing simulator (EGO, G) under and angle of 135 ° and the denture tooth of the prostheses was centrally loaded with a steatite ball (diameter = 6 mm) as antagonist (scheme see Fig. 2). The specimens were mechanically loaded (ML) with 50 N for 1,200,000 loadings with a frequency of 1.6 Hz. A simultaneous thermal cycling (TC) process was performed for 2 × 3000 cycles between 5 °C and 55 °C with deionized water. Each cycle lasted 2 min. The prostheses were monitored during the test and failure and the respective number of cycles (relative survival) was recorded.

Surviving specimens were loaded until failure in the comparable load situation as under TCML (Z10, Zwick, G) to determine the fracture force. The force was applied with a 6 mm thick steel ball with a tin foil between ball and prosthesis. The force was applied with velocity of 1 mm/min. The fracture pattern was classified with the help of a digital microscope (VHX-S550E; Keyence, G) differentiation for failures after TCML and after fracture test. Failures were divided into failure of the denture tooth (t), failure of the adhesive between base and tooth (a), fracture of the denture base (b), or mixed failure (m = a + b + t) (Fig. 2).

Calculations and statistical analysis were performed using SPSS 29.0 for Windows (SPSS Inc., Chicago, IL, USA). Distribution of the data was controlled with Shapiro-Wilk-test. Means and standard deviations were calculated and analyzed using one-way analysis of variance and the Bonferroni-test for post-hoc analysis. Kaplan-Meier survival and Pearson correlation were calculated. The level of significance was set to α = 0.05.

## Results

**Fig. 3:**
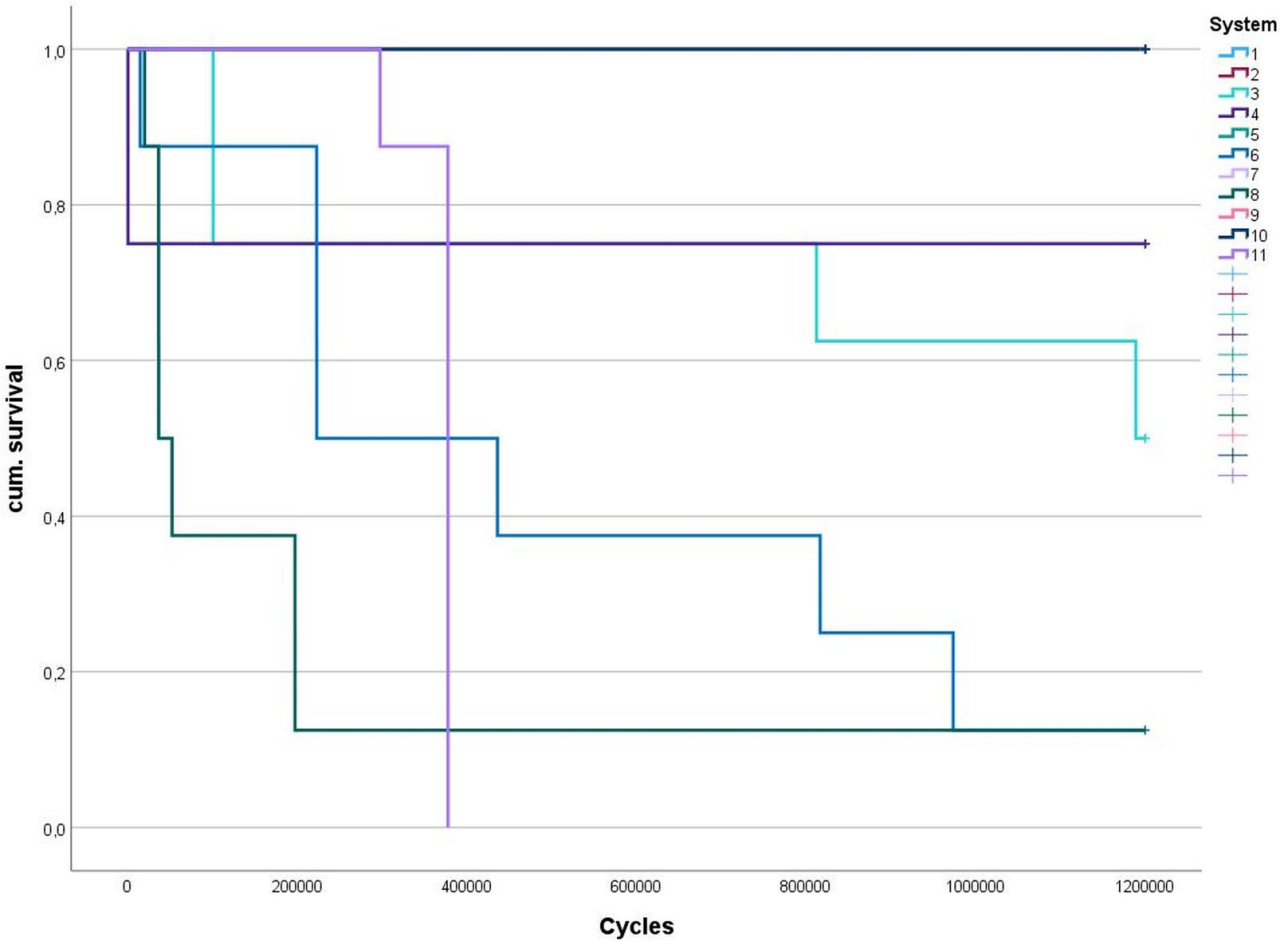
Kaplan-Meier survival (Log Rank Mantel-Cox test *p* < 0.001; Chi-Quadrat: 89.761, degree of freedom: 10)

Mean loading cycles until failure varied between 221,869 (8), 367,610 (11), 513,616 (6) 875,371 (3) and 9,000,030 (4). All specimens of groups 1, 2, 5, 7, 9 and 10 survived 1,200,000 cycles without failures. The number of dentures which failed during TCML ranged between 0 × (1, 2, 5, 7, 9, 10), 2 × (4), 4 × (3), 7 × (6, 8) and 8 × (11). Failure of the dentures during TCML (total *n* = 28) was characterized by failure of denture tooth (T, 2x), mixed base/tooth (M, 11x) or adhesive between base and tooth (A, 15x). ANOVA comparison revealed significant (*p* ≤ 0.001) differences in the number of surviving cycles between the individual systems. Individual significant (*p* < 0.036) differences were found (Table 2). Kaplan-Meier survival displays the loading cycles (Fig. 3). Log Rank (Mantel-Cox) test showed significantly (*p* < 0.001) different loading cycles between the systems (Chi-Quadrat: 89.761, degree of freedom: 10). Different (Bonferroni test) survival times (mean, standard deviation, minimum) and failure patterns are displayed in Fig. 3; Table 2.

**Table 2:** Loading cycles until failure during TCML (mean, standard deviation and minimum) and type of fracture (failure of denture base (B), denture tooth (T), mixed base/tooth (M) or adhesive between base and tooth (A); identical letters indicate statistically significant differences; α < 0.05)

	Loading cycles [number]			Fracture (*n*=)			
Code	Mean	Std	Min	T	A	M	B
1^abc^	1,200,000	0	1,200,000				
2^def^ 34	1,200,000	0	1,200,000				
3^gh^	875,317	496,337	100,564		4		
4^ij^	900,031	555,435	102		2		
5^klm^	1,200,000	0	1,200,000				
6^adknop^	513,617	428,193	14,532		5	2	
7^nqr^	1,200,000	0	1,200,000				
8^begilqst^	221,870	401,955	19,737		4	3	
9^osu^	1,200,000	0	1,200,000				
10^ptv^	1,200,000	0	1,200,000				
11^cfhjmruv^	367,610	28,315	297,533	2		6	
total				2	15	11	0

**Fig. 4:**
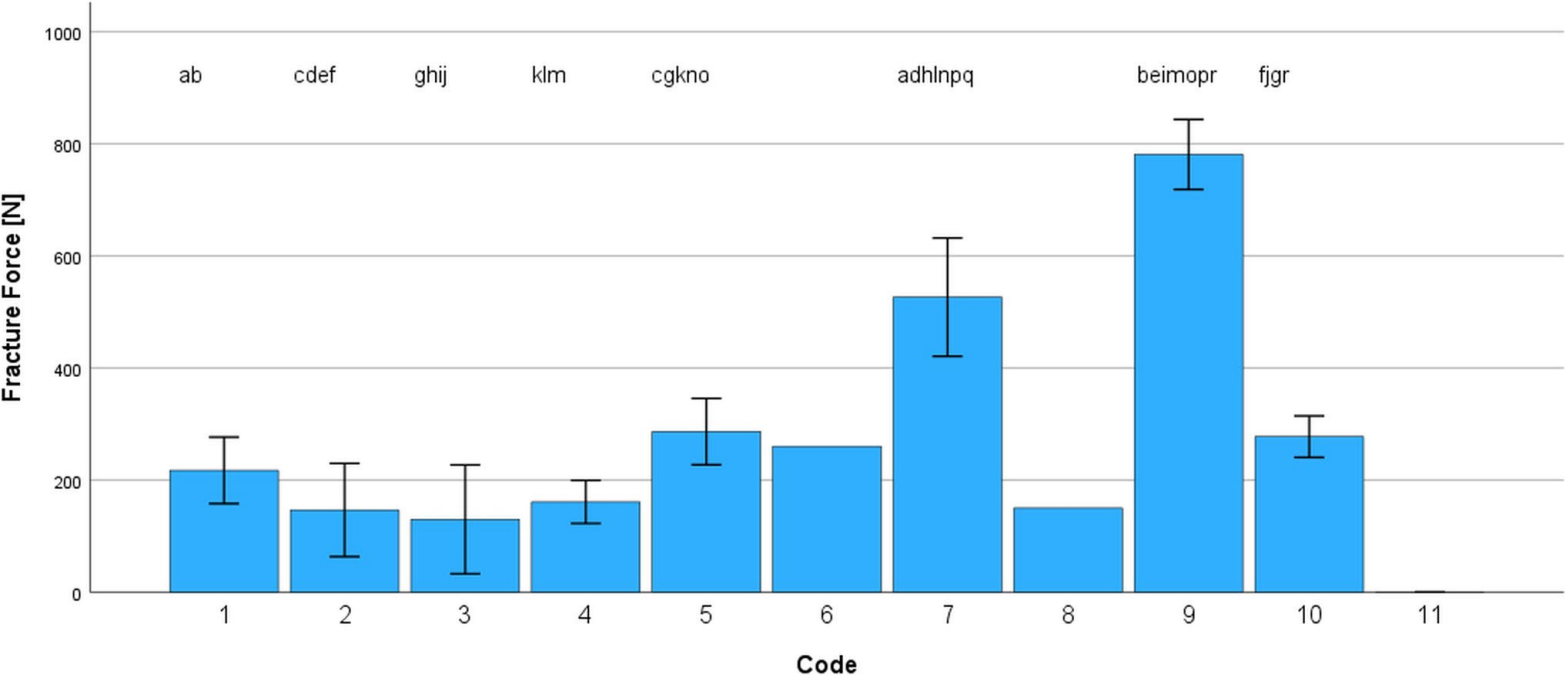
Fracture force ([N], mean and standard deviation) after TCML (identical letters indicate statistically significant differences; α < 0.05)

Mean fracture force after TCML varied between 129.8 +/- 97.1 N (3) and 780.8 +/- 62.5 N (9). Distribution of the fracture force was normal for all systems (*p* = 0.075–0.558). Due to the partly low survival numbers, an interpretation of the statistical fracture results should be done with care. ANOVA comparison revealed significant (*p* < 0.001) different fracture loadings between the individual systems, but individual significant (*p* < 0.049) differences were found (Table 3; Fig. 4). Failure of the dentures (total: *n* = 60) during load to fracture was characterized by failure of denture base (B, 2x), mixed base/tooth (M, 21x), adhesive (A, 14x) or denture tooth (T, 23x). Individual different (Bonferroni test) fracture loads and failure pattern are displayed in Table 3. A Pearson correlation was found between fracture force and loading cycles (0.587, *p* < 0.001).

**Table 3:** Type of fracture after fracture testing (failure of denture base (B), denture tooth (T), mixed base/tooth (M) or adhesive between base and tooth (A))

	Fracture (*n*=)			
Code	T	A	M	B
1		1	7	
2 3		3	4	1
3		4		
4		6		
5	8			
6			1	
7	7			1
8			1	
9	8			
10^r^			8	
11				
total	23	14	21	2

## Discussion

The hypothesis that dentures made from different teeth (milled, 3D-printed, fabricated), bases (milled, 3D-printed, pressed) and bonding combinations have different in-vitro survival rates and fracture resistance could be confirmed.

### Loading cycles until failure

The standard reference of direct polymerization of the prefabricated teeth and the adhesive bonding of refabricated teeth provided survival over the whole simulation period. The in-vitro results might reflect clinical data of pressed/ prefabricated dentures with clinical survival up to at least 3 years [15].

The influence of the base is shown, because the number of failures during simulation increased with the bonding of the prefabricated teeth to a 3D-printed base, and further with the use of printing resin as a bonding agent. Similar performance was found, when milled teeth were bonded to a pressed, milled or 3D-printed base. The results indicate that the bonding quality seems reduced in combination with 3D-printed bases, as further on also the number of adhesive and mixed failures increased. If the 3D-printed teeth were bonded to a 3D-printed base, the survival was significantly reduced even further [16].

Only with the simultaneous printing of tooth and base, no failures during TCML were found. Thus, the combined printing of tooth and base eliminated the bonding as a weak point of the denture.

These results also suggest that besides the bonding, the base type contributed relevantly to the survival of the dentures. In general, pressed or milled base materials seem advantageous in comparison to 3D-printed systems [17, 18]. Reason for the different performance might be a different flexural modulus of the materials [8, 9, 19–21]. However, it is not the strength of the base that seems to be decisive, but the ability to bond the tooth well to the base. It has been shown earlier, that milled or pressed systems may allow for a better bonding to the tooth [16]. This phenomenon was also evident in the high number of adhesive failures during simulation. Even clinical applications [15] provide similar low frequencies of a base fracture.

In conclusion, for the 3D-printed systems, only a simultaneously 3D-printed and aesthetically individualized denture might be used as long-term denture, whereas the other combinations with 3D-printed bases might be at least used as a try-in or emergency denture in the tested configurations [22–24]. 

The 3D-printed teeth showed even a negative influence on the performance of the complete denture, as only lower survival rates were observed in comparison to prefabricated or milled teeth. Tooth fracture during simulation was found only for 3D-printed teeth, which might be attributed to the layered fabrication of the tooth. Improvements might be achieved by using thinner layers, varying the building direction [25, 26] or by improving post-polymerization [27]. In contrast, the subsequent bonding of milled teeth into a milled base resulted in a good survival during the simulation process. Especially in case, if the denture was milled in one part, high survival rates could be observed. Less fabrication steps are required in this process, also reducing the susceptibility to fabrication errors [12, 28–30].

Specimens, which were processed according to the manufacturer’s instructions, generally showed the longest survival rates. If a denture is manufactured alternatively, it performs significantly worse [31, 32].

The analysis of different fracture patterns and survival rates during the simulation might help to further improve the fabrication of the dentures: Failures, which occurred right in the beginning of the aging procedure (around up to 20% of the maximum cycles), might be attributed to direct failures during the fabrication process. Here, the applied chewing force of 50 N was already sufficiently high to cause fracture of the denture. In contrast, failures occurring over the entire service life might be an indication of a system’s susceptibility to faults or might be an indication of aging effects.

### Load to fracture

Against expectations, the fracture force may play only a minor role for estimating a clinical performance: the conventional fabrication (prefabricated tooth and pressed base), which was used even as a clinical reference, showed fracture values only of about 150 N. Partly the surviving specimens of the 3D-printed combinations - with exception of group no. 11 – showed comparable values. In contrast, one-piece specimens displayed three to five times higher values under the same conditions.

In general, the load to fracture of the surviving specimens indicated a good to sufficient stability of the surviving milled and pressed dentures. Nevertheless, especially in combination with 3D-printed parts, the fracture forces were partially significantly lower. 3D-printed teeth showed mixed fracture pattern even at low loads, again indicating a material failure in the 3D-printed teeth. As seen above, the printing parameters might influence the quality of the teeth and, thus, their strength [25–27, 33]. However, studies show both worse [34] and comparable mechanical properties [35, 36] for 3D printed teeth in comparison to milled teeth.

The influence of the bonding on the fracture force was immanent: highest fracture values were found for dentures, which were fabricated as a single part in milling or - with a little bit lower values - in printing fabrication. These high fracture forces were always accompanied by a fracture of the tooth. Therefore it might be concluded that the weakest point of the denture might be again the bonding ability to the base material [37, 38]. Lowest fracture values, which were accompanied with mainly adhesive and mixed fracture patterns, might confirm these results and partly reflect the clinical situation [15]. It has been shown earlier that the type of bonding material might influence the bonding quality [39, 40]. In systems in which tooth, base and bonding were MMA based, mainly mixed fractures were characterized, whereas bonding with other resin and a 3D-printed base led to adhesive fractures. This was also shown by comparing milled materials from different companies [37].

The present study shows the influences of the different tooth-base combinations on the in-vitro performance of the restorations. The necessarily restricted selection of materials for teeth and base is certainly a limitation. Only one design of teeth with the corresponding bonding surface could be examined. Therefore, it is important to check whether the results can also be transferred to another tooth design or another tooth shape, e.g. in the posterior region with different types of loading. In clinical use, the thickness of the denture base might influence the overall stability and therefore must be considered during designing [41, 42]. It should also be noted that TCML is only a simulation of the clinical application, which is subject to the corresponding restrictions of such a time-lapsed aging procedure. In addition, the fracture tests simulate only a limited realistic load on the restorations, but allow to indicate the damage or defects caused by the simulation, particularly in the case of reduced forces. Although some systems showed a conclusively high failure force of the remaining test specimens, some also failed during TCML. This shows that some systems can achieve fundamentally stable values but are not consistently reliable.

## Conclusions

Strongly different performance was found for dentures made of different teeth (milled, 3D-printed, prefabricated), bases (milled, 3D-printed, pressed) and bonding combinations in terms of their survival rates and fracture forces. Milled, pressed and prefabricated systems provided longer survival and fracture force than the other tested systems. Optimal combinations can help to produce a denture that is stable and resistant to the effects of aging.

## Data Availability

No datasets were generated or analysed during the current study.
